# Vitamin and mineral status in chronic fatigue syndrome and fibromyalgia syndrome: A systematic review and meta-analysis

**DOI:** 10.1371/journal.pone.0176631

**Published:** 2017-04-28

**Authors:** Monica L. Joustra, Isidor Minovic, Karin A. M. Janssens, Stephan J. L. Bakker, Judith G. M. Rosmalen

**Affiliations:** 1 Interdisciplinary Center Psychopathology and Emotion regulation, University Medical Center Groningen, University of Groningen, Groningen, the Netherlands; 2 Department of Nephrology, University Medical Center Groningen, University of Groningen, Groningen, the Netherlands; 3 Top Institute Food and Nutrition, Wageningen, the Netherlands; Seoul National University, REPUBLIC OF KOREA

## Abstract

**Background:**

Many chronic fatigue syndrome (CFS) and fibromyalgia syndrome (FMS) patients (35–68%) use nutritional supplements, while it is unclear whether deficiencies in vitamins and minerals contribute to symptoms in these patients. Objectives were (1) to determine vitamin and mineral status in CFS and FMS patients as compared to healthy controls; (2) to investigate the association between vitamin and mineral status and clinical parameters, including symptom severity and quality of life; and (3) to determine the effect of supplementation on clinical parameters.

**Methods:**

The databases PubMed, EMBASE, Web of Knowledge, and PsycINFO were searched for eligible studies. Articles published from January 1^st^ 1994 for CFS patients and 1990 for FMS patients till March 1^st^ 2017 were included. Articles were included if the status of one or more vitamins or minerals were reported, or an intervention concerning vitamins or minerals was performed. Two reviewers independently extracted data and assessed the risk of bias.

**Results:**

A total of 5 RCTs and 40 observational studies were included in the qualitative synthesis, of which 27 studies were included in the meta-analyses. Circulating concentrations of vitamin E were lower in patients compared to controls (pooled standardized mean difference (SMD): -1.57, 95%CI: -3.09, -0.05; p = .042). However, this difference was not present when restricting the analyses to the subgroup of studies with high quality scores. Poor study quality and a substantial heterogeneity in most studies was found. No vitamins or minerals have been repeatedly or consistently linked to clinical parameters. In addition, RCTs testing supplements containing these vitamins and/or minerals did not result in clinical improvements.

**Discussion:**

Little evidence was found to support the hypothesis that vitamin and mineral deficiencies play a role in the pathophysiology of CFS and FMS, and that the use of supplements is effective in these patients.

**Registration:**

Study methods were documented in an international prospective register of systematic reviews (PROSPERO) protocol, registration number: http://www.crd.york.ac.uk/PROSPERO/display_record.asp?ID=CRD42015032528.

## Introduction

Chronic fatigue syndrome (CFS) and fibromyalgia syndrome (FMS) are syndromes of unknown origin. The core symptom of CFS is profound disabling fatigue [1], whereas FMS is characterized by chronic widespread pain [2,3]. CFS and FMS are known for substantial clinical and diagnostic overlap, for example, chronic pain and fatigue are common in both patient groups. The two syndromes are often comorbid; up to 80% of CFS patients reported a history of clinician-diagnosed FMS [4,5]. This has resulted in the hypothesis that these syndromes share etiological pathways [6].

Vitamin and mineral deficiencies may play a role in the pathophysiology of both CFS and FMS, although mechanisms behind this hypothesis are not entirely clear [7,8]. In addition, results of studies investigating the effects of nutritional supplementation or dietary intake on, for example, symptom severity in these patient groups, are conflicting [9–12]. Nevertheless, a large proportion of CFS and FMS patients indicate they use nutritional supplements (35%-68%) [10,13–15], compared to the Dutch general population (27–56%) [16]. The higher nutritional supplement use among patients may be due to encouragements by specialty stores, the internet or (complementary medicine) clinics. Vitamins and minerals in these products are sometimes supplemented in doses high enough to cause health problems, for example gastric discomfort, insomnia, dizziness and weakness [17]. More information is needed on the evidence for (marginal) vitamin and mineral deficiencies in CFS and FM, and the potential benefits in taking nutritional supplements.

Recently, a review investigating hypovitaminosis D in both chronic pain and FMS patients showed that these patients were at significantly higher risk of hypovitaminosis D than healthy controls [18]. Unfortunately, further reviews on vitamin and mineral deficiencies among CFS and FMS patients are lacking. We therefore carried out this first systematic review on vitamin and mineral status in CFS and FMS. We explored the following research questions: first, what is the evidence for deficiencies in vitamin and mineral status in CFS and FMS patients as compared to healthy controls? Second, is vitamin and mineral status associated with clinical parameters, including symptom severity and quality of life, in CFS and FMS? Third, what is the evidence for an effect of vitamin and mineral supplementation, as compared to placebo, on clinical parameters in CFS and FMS patients? Because it is currently unknown whether CFS and FMS result from the same etiology, we analyzed results both for the combined and for the separate syndromes.

## Methods

We followed the Preferred Reporting Items for Systematic Reviews and Meta-Analyses (PRISMA) guidelines (S1 Table) [19]. Prior to start of article inclusion, we documented study methods in an international prospective register of systematic reviews (PROSPERO) protocol, registration number: CRD42015032528, http://www.crd.york.ac.uk/PROSPERO/display_record.asp?ID=CRD42015032528.

### Data sources and searches

The databases PubMed, EMBASE, Web of Knowledge, and PsycINFO were systematically searched. Articles published between January 1^st^ 1994 and 1990, for CFS and FMS respectively, and March 1^st^ 2017 were included. We focused on the most recent diagnostic guidelines, namely the International Center of Disease Control (CDC) diagnostic criteria for CFS that was established in 1994 [1], and the American College of Rheumatology (ACR) criteria for FMS in 1990 [2]. To retrieve relevant articles from PubMed, we formulated a search string (S1 Appendix) that consisted of CFS, FMS, and synonyms, vitamins, minerals, micronutrients and synonyms, while excluding systematic reviews or animal studies. This search string was adapted according to the thesaurus of the databases EMBASE, Web of Knowledge, and PsycINFO. All included studies were screened for potential references that were not included in the first search. Duplicates were removed, as well as studies including pediatric participants. There were no language restrictions; included non-English articles were translated (French, Italian, Polish, and Turkish articles) by native speakers.

### Study selection

Title and abstract were screened by two independent reviewers (M.L.J. and I.M.) for the following criteria: (1) CFS or FMS patients; (2) vitamin or mineral status; and (3) study design. Studies which were in agreement with the eligibility criteria were retrieved as full text. Discrepancies between the two researchers were resolved by consensus, and when needed a third assessor was consulted (J.G.M.R.). Reasons for exclusion and percentage of agreement, as Cohen’s kappa, between the assessors were documented.

Participants of the included studies had to be adults (i.e. ≥18 years) suffering from CFS or FMS according to the official diagnostic criteria [1–3]. Studies that involved patients with a combination of CFS and FMS or other comorbid medical conditions were excluded. Furthermore, the vitamin or mineral status had to be assessed or reported in the article, or there had to be an intervention concerning vitamins or minerals. Patients were compared with healthy controls in observational studies, or vitamin and mineral supplementation were compared with placebo in intervention studies. Lastly, cross-sectional studies comparing cases and controls, cohort studies and randomized controlled trials (RCTs) were included. Case reports, clinical cohorts without appropriate controls (e.g. controls with musculoskeletal pain or fatigue), (systematic) reviews, expert opinion, and other study designs were excluded.

### Data extraction

Two reviewers (M.L.J. and I.M.) independently extracted data and assessed the risk of bias for each study. The first ten articles were screened together to pilot the data extraction and risk of bias form. Reasons for exclusion and percentage of agreement between the assessors were documented.

From the included articles, the following information was extracted: name first author, publication year, type FSS, number and age of the participants, and vitamin or mineral status. In addition, data on smoking habits or alcohol use, diet (and assessment tool used), BMI (or waist circumference, waist-hip ratio), physical activity (assessment tool), socioeconomic status, ethnicity, severity of illness (assessment tool), duration of illness, co-morbidities (somatic and psychiatric), medication use, clinical parameters including symptom severity and quality of life, and in case of RCTs the relevant co-intervention(s) were also extracted.

### Quality assessment

To assess quality of RCTs, the Cochrane Collaboration’s tool for assessing risk of bias was employed [20]. For observational studies, literature indicates lack of a single methodological assessment tool [21,22]. Therefore, we adjusted a previously developed quality tool for observational studies in this field [23], for use in studies that focus specifically on the association between vitamin and mineral status and CFS or FMS. Eight of the nine items in this original quality tool originated from guidelines or tools for either reporting or appraising observational research [24–26]. These items were adjusted to the specific question on vitamins and minerals and classified into three key domains: appropriate selection of participants (validated disorder, representative controls, in- and exclusion criteria, disease characteristics), appropriate quantification of vitamin and mineral status (duplicate quantification, appropriate outcome), and appropriate control for confounding (assessed confounders, analyses adjusted). The item: “Is the assessor blind for disease status”, was excluded since from the original quality tool since it is not applicable in the current review. Furthermore, we added the item “Are methods for assessment of vitamin and mineral status clearly stated”, based on the adapted Newcastle Ottawa scale for cross-sectional studies (S2 Appendix) [27]. RCTs that contained relevant observational data (n = 4/5), were assessed with both the Cochrane tool and the observational studies quality tool. For both quality tools, items were rated as (0) low risk, (1) medium risk, and (2) high risk of bias. The maximum attainable quality score was 14 for RCTs, and 18 for observational studies.

### Data synthesis and analysis

We first constructed an overview of available data on the different vitamins and minerals. Characteristics of the included studies were systematically listed to generate a clear overview of the current literature on vitamins and minerals in CFS and FMS patients. For those vitamins and minerals with more than five studies available, we did quantitative syntheses on aggregated data. For these syntheses, data was pooled with the random effects model of meta-analysis, using Stata statistical software, version 14 (Statacorp LP, Texas). To allow pooling across studies that used different outcomes of vitamin or mineral plasma or serum levels, we calculated the standardized mean difference (SMD). For proportions of deficiencies, the odds ratio (OR) was calculated and pooled. Subsequently, the SMD and OR for each study were weighted by their inverse variance and the corresponding 95%CI were calculated. The existence of heterogeneity among studies was assessed by Q-tests, and the degree of the heterogeneity was quantified by calculating the I-squared (I^2^) value. Publication bias was inspected visually by a funnel plot, and an Egger’s test was conducted to quantify funnel plot asymmetry [28]. The Tweedie’s Trim and Fill test was performed as an additional sensitivity analysis to identify and correct for funnel plot asymmetry arising from publication bias [29]. When the Trim and Fill test was performed, and additional studies were added to the analyses, contour-enhanced funnel plots were used instead of regular funnel plots to examine whether asymmetry in the funnel plots was due to publication bias [30]. Subgroup analyses were performed including studies with more than half of the maximum study quality score (>9 quality points), if more than three studies with a sufficient quality score were available. Furthermore, vitamin and mineral status of CFS and FMS patients were investigated separately if more than three studies were available. Findings were considered statistically significant if P<0.05.

## Results

### Study inclusion

**Fig 1:**
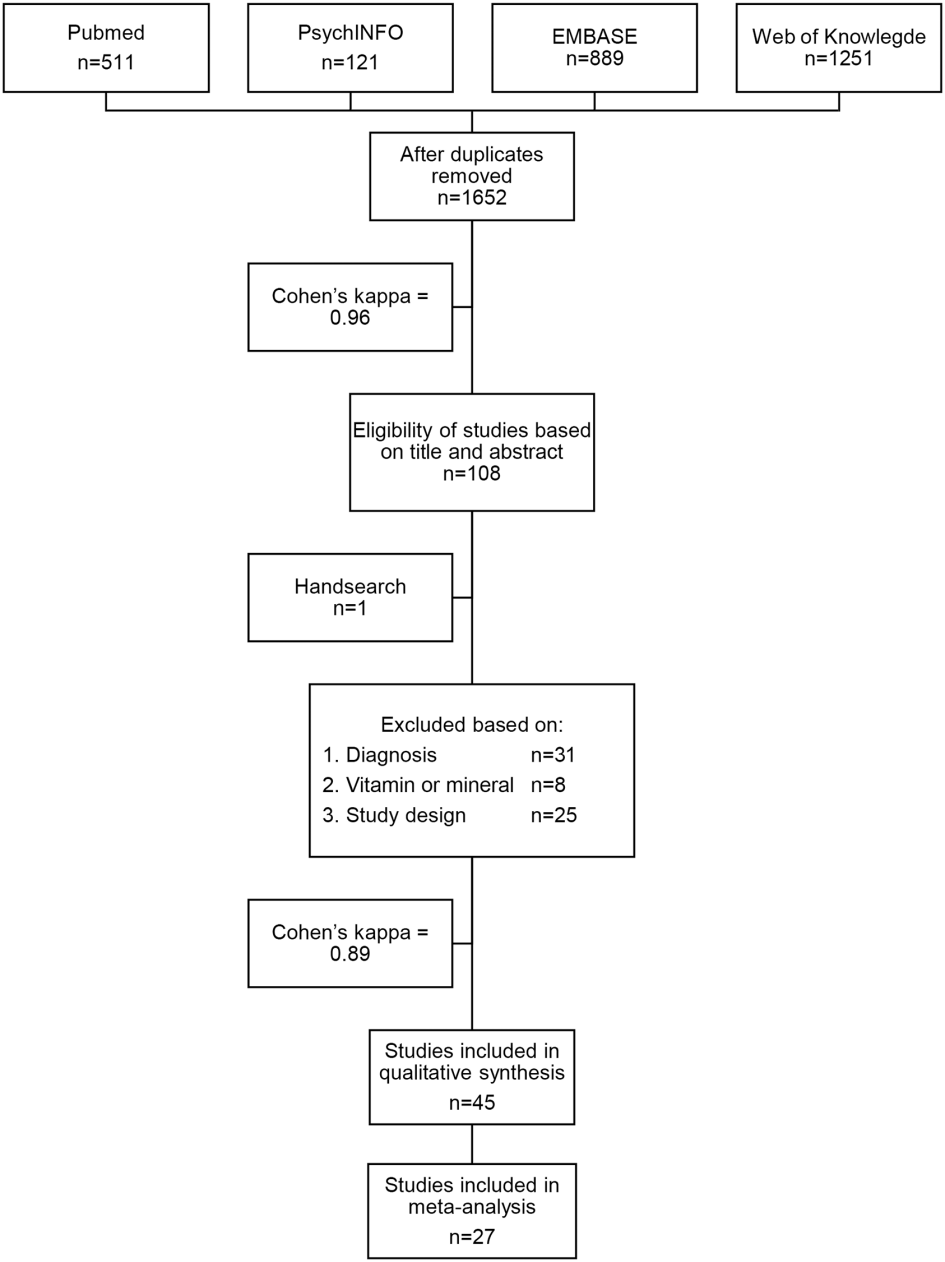
Flow diagram.

Results of the systematic review and meta-analysis are presented in a flow diagram (Fig 1). Cohen’s kappa’s for the abstract and full text selection were 0.96 and 0.89 respectively, indicating very good consistency of agreement [31]. Out of 108 studies included for the full text review, 45 studies were included in the current review.

Characteristics of the included studies are presented in Table 1, and results of the quality assessment in Tables 2 and 3. Most studies involved FMS patients (n = 35/45); 4 of the 5 RCTs also contained relevant observational data. Vitamin and mineral status was mainly assessed in plasma or serum (n = 40/45). Furthermore, quality scores revealed poor study quality (i.e. equal or less than half of the maximum study quality score) in the vast majority of observational studies (n = 27/44; range 4–14 points) and RCTs (n = 3/5; range 5–12 points). Only few observational studies defined all described in- and exclusion criteria for the investigated population, including medication use, somatic morbidity, and psychiatric morbidity (n = 10/44). The CFS or FMS diagnostic criteria were often described in observational studies, but researchers failed to state whether or not the syndromes were diagnosed by a physician (n = 40/44). Disease characteristics were frequently not fully presented (n = 15/44), or were completely absent (n = 18/44) in observational studies. Almost all observational studies did not assess vitamin or mineral in duplicate (n = 38/44). Most studies that assessed vitamin or mineral status did not clearly state the methods for assessment of vitamin and mineral status (n = 27/44). Furthermore, most observational studies did not adjust their analyses for any potential confounders (n = 43/44). Lastly, most RCTs had a medium to high risk of bias for random sequence generation (n = 3/5), allocation concealment (n = 3/5), blinding of outcome assessment (n = 4/5), incomplete data (n = 4/5), selective reporting quantification (n = 3/5), and other bias (n = 5/5).

**Table 1:** Characteristics of included studies.

**Study**	**Setting**	**Type of FSS**	**N of cases**	**Study design**	**Mean age in years (SD)**	**Mean FSS severity (SD) and/or mean duration in months (SD)**	**Comparison group (n)**	**Vitamin and/or mineral**	**Material**
**Akkus et al, 2009** [32]	Secondary care	FMS	30	Case-control	40.1 (5.2)	FIQ: 59.8 (7.9) 68.8	Healthy controls (30)	Vitamin A, C, E	Plasma
**Al-Allaf et al, 2003** [33]	Secondary care	FMS	40	Case-control	42.5 (3.6)	FIQ (score out of 10): 6.5 (2.2) 48 (31)	Healthy controls (37)	Vitamin D, calcium	Serum
**Bagis et al, 2013** [34]	Secondary care	FMS	60	RCT and case-control	40.7 (5.2)	FIQ: 38.8 (10.4)	Healthy controls (20)	Magnesium	Serum, erythrocytes
**Baygutalp et al, 2014** [35]	Secondary care	FMS	19	Case-control	35 (7.5)	FIQ: 19.3 (21.5) 4.4 (1.2)	Healthy controls (21)	Vitamin D	Serum
**Bazzichi et al, 2008** [36]	Secondary care	FMS	25	Case-control	48.8 (9.3)	FIQ: 57.9 (17.3)	Secondary care patients without FM or musculo-skeletal pain (25)	Calcium, magnesium	Platelets
**Brouwers et al, 2002** [37]	Tertiary care	CFS	24	RCT	40.0 (9.9)	CIS: 51.4 (4.2) Disease duration (years, median (IQR)) 8.0 (2–15)	Placebo, CFS patients (25)	Polynutrient supplement	NA
**Costa et al, 2016** [38]	Secondary care	FMS	100	Case-control	42.4 (8.4)	NR	Healthy controls (57)	Calcium	Serum
**Eisinger et al, 1997** [39]	NR	FMS	25	Case-control	40	NR	Healthy controls (20)	Vitamin A, E, magnesium, selenium, zinc	Plasma
**Eisinger et al, 1996** [40]	NR	FMS	25	Case-control	40	NR	Healthy controls (20)	Magnesium	Serum, erythrocytes, lencocytes
**Study**	**Setting**	**Type of FSS**	**N of cases**	**Study design**	**Mean age in years (SD)**	**Mean FSS severity (SD) and/or mean duration in months (SD)**	**Comparison group (n)**	**Vitamin and/or mineral**	**Material**
**Heidari et al, 2010** [41]	Secondary care	FMS	17	Case-control	40.6 (8.3)	NR	Secondary care patients without FM or musculoskeletal pain (202)	Vitamin D	Serum
**Jammes et al, 2011** [42]	NR	CFS	5	Case-control	39 (8)	72 (12)	Healthy controls (23)	Vitamin C, potassium, sodium	Plasma
**Jammes et al, 2009** [43]	Secondary care	CFS	18	Case-control	38 (5)	NR	Medical checkup patients (9)	Vitamin C	Plasma
**Kasapoğlu Aksoy et al, 2016** [44]	Secondary care	FMS	53	Case-control	48.2 (9.6)	VAS pain (0–10) median, min-max: 8.0 (4.0–10.0)	Healthy controls (47)	Vitamin D	Serum
**Khalifa et al, 2016** [45]	Secondary care	FMS	31	Case-control	40.2 (13.3)	FIQR mean: 32.4	Healthy controls (21)	Calcium, copper, magnesium, zinc	Serum
**Kim et al, 2011** [46]	Secondary care	FMS	44	Case-control	42.5 (6.9)	NR	Healthy controls (122)	Calcium, copper, ferritin, magnesium, manganese, phosphorus, potassium, selenium, sodium, zinc	Hair
**Kurup et al, 2003** [47]	Secondary care	CFS	15	Case-control	30–40 range	NR	Healthy controls (15)	Vitamin E, magnesium	Plasma, RBC
**La Rubia et al, 2013** [48]	NA	FMS	45	Case-control	52.2 (7.5)	FIQ: 61.4 (13.1)	Healthy controls (25)	Copper, ferritin, iron, zinc	Serum
**Maafi et al, 2016** [49]	Tertiary care	FMS	74	Case-control	37.9 (9.8)	FIQR: 51.8 (17.2) 13.2 (6.2)	Healthy controls (68)	Vitamin D, calcium, phosphorus	Serum
**Mader et al, 2012** [50]	Secondary care	FMS	84	Case-control	52 (12)	FIQ: 57.1 (20.2)	Healthy controls (87)	Ferritin, iron	Serum
**Maes et al, 2006** [51]	Secondary care	CFS	12	Case-control	41.9 (13.2)	NR	Healthy controls (12)	Zinc	Serum
**Study**	**Setting**	**Type of FSS**	**N of cases**	**Study design**	**Mean age in years (SD)**	**Mean FSS severity (SD) and/or mean duration in months (SD)**	**Comparison group (n)**	**Vitamin and/or mineral**	**Material**
**Mateos et al, 2014** [52]	Secondary care	FMS	205	Case-control	51.5 (9.6)	NR	Healthy controls (205)	Vitamin D, calcium	Serum
**McCully et al, 2005** [53]	NR	CFS	20	Case-control	NR	NR	Healthy sedentary controls (11)	Magnesium	Skeletal muscle
**Mechtouf et al, 1998** [54]	NR	FMS	54	Case-control	Min-max: 20–75	NR	Healthy controls (36)	Vitamin B1	Plasma
**Miwa et al, 2010** [55]	Secondary care	CFS	27	Case-control	29 (6)	NR	Secondary care patients free from fatigue for at least a month (27)	Vitamin E	Serum
**Miwa et al, 2008** [56]	NR	CFS	50	Case-control	NR	NR	Healthy controls (40)	Vitamin E	Serum
**Nazıroğlu et al, 2010** [57]	Secondary care	FMS	31	RCT and case-control	40.1 (5.2)	Number tender points: 15 (2)	Healthy controls (30)	Vitamin A, C, E	Plasma
**Ng et al, 1999** [58]	Secondary care	FMS	12	Case-control	44.6	NR	Healthy controls (12)	Calcium, magnesium	Hair
**Norregaard et al, 1994** [59]	NR	FMS	15	Case-control	49	NR	Healthy controls (15)	Potassium	Plasma
**Okyay et al, 2016** [60]	Tertiary care	FMS	79	Case-control	37 (9)	NR	Healthy controls (80)	Vitamin D	Serum
**Olama et al, 2013** [61]	Secondary care	FMS	50	Case-control	32.3 (9.4)	47 (24)	Healthy controls (50)	Vitamin D, calcium, phosphorus	Serum
**Ortancil et al, 2010** [62]	Secondary care	FMS	46	Case-control	46.9 (10.6)	FIQ: 60.0 (10.9)	Healthy controls (46)	Vitamin B12, ferritin, folic acid	Serum
**Özcan et al, 2014** [63]	Secondary care	FMS	60	Case-control	41.9 (9.8)	FIQ: 58.6 (10.3) 27.3 (17.3)	Healthy controls (30)	Vitamin D	Serum
**Study**	**Setting**	**Type of FSS**	**N of cases**	**Study design**	**Mean age in years (SD)**	**Mean FSS severity (SD) and/or mean duration in months (SD)**	**Comparison group (n)**	**Vitamin and/or mineral**	**Material**
**Reinhard et al, 1998** [64]	Secondary care	FMS	68	Case-control	47	NR	Blood donors without FM or musculoskeletal pain (97)	Selenium	Serum
**Rezende Pena et al, 2010** [65]	Secondary care	FMS	87	Case-control	44.9 (8.6)	Number tender points: 14 (5)	Secondary care patients without FM or musculoskeletal pain (92)	Vitamin D	Serum
**Rosborg et al, 2007** [66]	Secondary care	FMS	38	Case-control	Median (min-max): 49 (31–71)	NR	Healthy controls (41)	Calcium, copper, ferritin, iodine, magnesium, molybdenum, potassium, selenium, sodium, zinc	Whole blood, fasting urine
**Sakarya et al, 2011** [67]	NR	FMS	40	Case-control	33.6 (7.6)	FIQ: 61.3 (9.2)	Healthy controls (40)	Vitamin A, C, E, magnesium	Plasma
**Samborski et al, 1997** [68]	Secondary care	FMS	60	Case-control	46,4 (9.8)	NR	Healthy controls (20)	Calcium	Plasma
**Sendur et al, 2008** [69]	NR	FMS	32	Case-control	42.9 (7.7)	FIQ: 53.3 (7.9)	Healthy controls (32)	Magnesium, selenium, zinc	Serum
**Tandeter et al, 2009** [70]	Secondary care	FMS	68	Case-control	43.8 (7.6)	NR	Regular periodic blood tests patients with no FM (82)	Vitamin D	Serum
**Türkyilmaz et al, 2010** [71]	Secondary care	FMS	30	Case-control	39.8 (6.2)	SF- 36: 47.4 (17.3) 72 (62.2)	Healthy controls (30)	Vitamin D, calcium, phosphorus	Serum
**Ulusoy et al, 2010** [72]	NR	FMS	30	Case-control	32.2 (6.8)	FIQ: 64.7 (14.3) 32.7 (19.7)	Healthy controls (30)	Vitamin D, calcium, phosphorus	Serum
**Study**	**Setting**	**Type of FSS**	**N of cases**	**Study design**	**Mean age in years (SD)**	**Mean FSS severity (SD) and/or mean duration in months (SD)**	**Comparison group (n)**	**Vitamin and/or mineral**	**Material**
**Vecchiet et al, 2002** [73]	Secondary care	CFS	21	Case-control	42 (8)	VAS muscle fatigue (0–100): 52.9 (4.9) 44.5 (27.6)	Healthy controls (20)	Vitamin E	Plasma, LDL
**Wepner et al, 2014** [74]	General population and secondary care	FMS	15	RCT and cross-sectional	Overall (n = 30) 48.3 (5.3)	Number tender points: 15 (2)	Placebo, FMS patients (15)	Vitamin D	Serum
**Witham et al, 2015** [75]	Secondary care	CFS	25	RCT and case-control	48.1 (12.0)	Piper fatigue scale: 6.3 (1.6)	Placebo, CFS patients (25)	*RCT*: depending on serum levels 2400 or 1200 IU cholecalciferol *Observational*: Vitamin D	Serum
**Yildirim et al, 2016** [76]	NR	FMS	99	Case-control	49.4 (9.2)	FIQ: 62.9 (17.7)	Healthy controls (99)	Vitamin D	Serum

CFS = chronic fatigue syndrome, CIS = checklist individual strength (8–56), FIQ = fibromyalgia impact questionnaire (0–100), FIQR = revised fibromyalgia impact questionnaire (0–100), FMS = fibromyalgia syndrome, FSS = functional somatic syndrome, NR = not reported, RBC = red blood cells, RCT = randomised controlled trail, VAS = visual analogue scale.

**Table 2:** Results of the quality assessment of observational studies.

	Appro-priate selection of par-ticipants	Validated disorder	Repre-sentative controls	In- and exclusion criteria	Disease charac-teristics	Appro-priate quanti-fication	Validated methods	Duplicate quanti-fication	Appro-priate outcome	Appro-priate control for con-founding	Assessed con-founders	Analyses adjusted	Total score
Akkus et al, 2009 [32]													10
Al-Allaf et al, 2003 [33]													9
Bagis et al, 2013 [34]													7
Baygutalp et al, 2014 [35]													14
Bazzichi et al, 2008 [36]													10
Costa et al, 2016 [38]													6
Eisinger et al, 1997 [39]													8
Eisinger et al, 1996 [39]													7
Heidari et al, 2010 [41]													8
Jammes et al, 2011 [42]													10
Jammes et al, 2009 [43]													11
Kasapoğlu Aksoy et al, 2016 [44]													8
Khalifa et al, 2016 [45]													6
Kim et al, 2011 [46]													9
Kurup et al, 2003 [47]													8
La Rubia et al, 2013 [48]													9
Maafi et al, 2016 [49]													11
Mader et al, 2012 [50]													9
Maes et al, 2006 [51]													8
Mateos et al, 2014 [52]													7
McCully et al, 2005 [53]													4
Mechtouf et al, 1998 [54]													6
Miwa et al, 2010 [55]													9
Miwa et al, 2008 [56]													6
Nazıroğlu et al, 2010 [57]													9
Ng et al, 1999 [58]													6
Norregaard et al, 1994 [59]													5
Okyay et al, 2016 [60]													8
Olama et al, 2013 [61]													11
Ortancil et al, 2010 [62]													10
Özcan et al, 2014 [63]													9
Reinhard et al, 1998 [64]													7
Rezende Pena et al, 2010 [65]													11
Rosborg et al, 2007 [66]													9
Sakarya et al, 2011 [67]													10
Samborski et al, 1997 [68]													4
Sendur et al, 2008 [69]													10
Tandeter et al, 2009 [70]													11
Türkyilmaz et al, 2010 [71]													10
Ulusoy et al, 2010 [71]													10
Vecchiet et al, 2002 [73]													10
Wepner et al, 2014 [74]													10
Witham et al, 2015 [75]													14
Yildirim et al, 2016 [76]													8
Total score mean (SD): 8.7 (2.2)													

According to the quality tool to assess methodological quality of vitamin and mineral studies in CFS and FM (S2 Appendix).

white = low risk, light gray = medium risk, dark gray = high risk

**Table 3:** Results of the quality assessment of randomized controlled trials.

	Random sequence generation	Allocation concealment	Blinding of participants and personnel	Blinding of outcome assessment	Incomplete data	Selective reporting quantification	Other bias	Total score
Bagis et al, 2013 [34]								5
Brouwers et al, 2002 [37]								6
Nazıroğlu et al, 2010 [57]								6
Wepner et al, 2014 [74]								8
Witham et al, 2015 [75]								12
Total score mean (SD): 10.0 (2.6)								

According to the Cochrane Collaboration’s tool.

white = low risk, light gray = medium risk, dark gray = high risk

### Systematic review

Studies that were not included in the meta-analyses are presented in Table 4.

**Table 4:** Vitamin and mineral status in the included studies.

**Vitamin A**						
**Study**	**Patients**		**Controls**		**Statistically significant**	**Linked to clinical parameter**
	**Mean**	**SD**	**Mean**	**SD**		
**Akkus et al, 2009** [32]	0.30 μmol/l	0.10	0.45	0.16	p<.01	NR
**Eisinger et al, 1997** [39]	2.7 μmol/l	1.5	2.3	0.9	NS	NR
**Nazıroğlu et al, 2010** [57]	1.5 μmol/l	0.5	2.4	0.2	p<.05	NR
**Sakarya et al, 2011** [67]	1.46 mmol/l	0.47	1.25	0.26	NS	*FIQ* *Pearson’s correlation coefficient*: -0.083 (NS)
**Vitamin B1**						
**Mechtouf et al, 1998** [54]	58 ng/ml	38.9	49.6	14.8	p<.05	NR
**Vitamin B12**						
**Ortancil et al, 2010** [62]	297.6 pg/ml	120.7	295.7	113.0	NS	NR
**Vitamin C**						
**Sakarya et al, 2011** [67]	x	x	x	x	x	*FIQ* *Pearson’s correlation coefficient*: -0.115 (NS)
**Vitamin D**						
**Al-Allaf et al, 2003** [33]	<20nmol/l (n (%)):	18 (45)	n (%):	7 (18.9%)	p<0.015	NR
**Baygutalp et al, 2014** [35]	x	x	x	x	x	*FIQ* *Spearman correlation*: 0.231 (NS)
**Kasapoğlu Aksoy et al, 2016** [44]	x	x	x	x	x	*<30 ng/ml vs >30 ng/ml in FMS*: *VAS pain*: 8.4 (1.6) *vs* 6.7 (2.0) p = .002 *FIQ*: 65.4 (12.0) *vs* 57.2 (16.1) p = .088
**Maafi et al, 2016** [49]	x	x	x	x	x	*FIQR* *Spearman correlation*: -0.093 (NS) *Number of tender points*: -0.194 (NS) *VAS pain*: -0.097 (NS)
**Okyay et al, 2016** [60]	x	x	x	x	x	*<20 ngl/ml vs 20–30 vs >30 ng/ml in FMS*: *FIQ*: 56.6 (8.9) *vs* 48.8 (2.8) *vs* 41.4 (8.2) p = .000 *VAS pain*: 7.4 (1.4) *vs* 6.4 (0.5) *vs* 5.1 (1.0) p = .000 *FIQ* *Spearman correlation*: -0.621 (p = .000) *VAS pain* *Spearman correlation*: -0.578 (p = .000)
**Study**	**Patients**		**Controls**		**Statistically significant**	**Linked to clinical parameter**
	**Mean**	**SD**	**Mean**	**SD**		
**Rezende Pena et al, 2010** [65]	x	x	x	x	x	*Number of tender points* *Pearson’s correlation coefficient*: -0.160 (NS) *VAS pain*: -0.196 (NS)
**Ulusoy et al, 2010** [72]	<20ng/l (n (%)):	26 (86.7)	n (%):	29 (96.7)	NS	*FIQ* *Pearson’s correlation coefficient*: 0.071 (NS)
**Wepner et al, 2014** [74]	19.94 ng/ml	6.066	NR	NR	NR	NR
**Witham et al, 2015** [75]	44 and 48 nmol/l	15 and 20	NR	NR	NR	*Piper fatigue scale*: no improvement after vitamin D3 treatment
**Yildirim et al, 2016** [76]	x	x	x	x	x	*FIQ* *Pearson’s correlation coefficient*: r = 0.112 (NS) *VAS pain*: r = 0.104 (NS)
**Vitamin E**						
**Kurup et al, 2003** [47]	5.22 μg/ml RBC	0.31	5.25	0.33	NS	NR
**Miwa et al, 2010** [55]	2.81 mg/g lipids	0.73	3.88	0.65	p<.001	NR
**Miwa et al, 2008** [56]	3.03 mg/g lipids	0.72	3.78	0.66	p<.001	NR
**Sakarya et al, 2011** [67]	x	x	x	x	x	*FIQ* *Pearson’s correlation coefficient*: −0.171 (NS)
**Vecchiet et al, 2002** [73]	9.5 μmol/mg LDL	1.0	18.0	1.5	p<.001	*Linear regression analyses* *fatigue* *versus vitamin E in plasma*: Y = 56.674–0.4467X r = -0.6098 (p < 0.004)
**Calcium**						
**Bazzichi et al, 2008** [36]	231.0 nM platelet	13.75 (SEM)	198.3	10.40	NS	NR
**Kim et al, 2011** [46]	775 μg/g	439–1,366 (95%CI)	1,093	591–2,020	p = .001	NR
**Ng et al, 1999** [58]	2288.4 μg/g hair	1486.2	846.3	645.7	p = .025	NR
**Rosborg et al, 2007** [66]	49 mg/l (median whole blood) 72.8 mg/l (median urine)	28.5–62.2 <29–258 (range)	48.0 74.5	39.7–58.5 <29–519	NS	NR
**Copper**						
**Khalifa et al, 2016** [45]	145.8 μg/dl	17.34	116.50	14.35	p<.05	NR
**Kim et al, 2011** [46]	28.3 μg/g	11.8–68.1 (95%CI)	40.2	16.1–100.0	p = .029	NR
**La Rubia et al, 2013** [48]	105.99 mg/dl	17.03	83.55	9.20	p<.001	NR
**Study**	**Patients**		**Controls**		**Statistically significant**	**Linked to clinical parameter**
	**Mean**	**SD**	**Mean**	**SD**		
**Rosborg et al, 2007** [66]	971 μg/l (median whole blood) 28.1 μg/l (median urine)	620–1740 6.7–186 (range)	855 34.7	690–1475 8.6–92.2	p = .002 NS	NR
**Ferritin**						
**Kim et al, 2011** [46]	5.90 μg/g	4.21–8.26 (95%CI)	7.10	4.73–10.66	p = .007	NR
**La Rubia et al, 2013** [48]	52.33 g/dl	15.07	57.42	17.01	NS	NR
**Mader et al, 2012** [50]	63.68 ng/ml ≤30 ng/mL n (%): 23 (27.4)	49.72	53.70 n (%): 38 (43.7)	46.24	p = .18 p<.04	*FIQ* *Spearman correlation*: NS
**Ortancil et al, 2010** [62]	27.3 ng/ml <50 ng/mL n (%): 40 (87.0)	20.9	43.8 n (%): 26 (56.5)	30.8	p = .035 p = .001	*FIQ* *Spearman correlation*: NS
**Rosborg et al, 2007** [66]	422 mg/l (median)	245–585 (range)	400	273–465	p = .046	NR
**Folic acid**						
**Ortancil et al, 2010** [62]	9.2 ng/ml	3.1	8.9	2.5	NS	NR
**Iodine**						
**Rosborg et al, 2007** [66]	<650 μg/l (median whole blood) 788 μg/l (median urine)	<650–1900 <130–5395 (range)	<650 2000	<650–693 <130–12145	NS p = .001	NR
**Iron**						
**La Rubia et al, 2013** [48]	81.82 mg/dl	34.64	83	30.07	NS	NR
**Mader et al, 2012** [50]	82.32 μg/dl	32.75	75.31	29.13	NS	*FIQ* *Spearman correlation*: NS
**Magnesium**						
**Bagis et al, 2013** [34]	Erythrocyte: 2.27/2.70/2.91 mmol/l	0.41/0.47/0.42	3.22 mmol/l	0.36	p<.001	*FIQ* *Pearson’s correlation serum Mg*: -0.426 (p<.001) *Erythrocyte Mg*: -0.309 (p = .013)
**Bazzichi et al, 2008** [36]	1.30 mM platelet	0.079 (SEM)	1.07	0.056	p = .02	NR
**Eisinger et al, 1997** [39]	2.36 mmol/l erythrocyte	0.24	2.39	0.24	NS	NR
**Eisinger et al, 1996** [40]	4.9 fmol/cell lencocyte	1.7	3.9	1.3	NS	NR
**Kim et al, 2011** [46]	52 μg/g	25–107 (95%CI)	72	36–147	p = .008	NR
**Study**	**Patients**		**Controls**		**Statistically significant**	**Linked to clinical parameter**
	**Mean**	**SD**	**Mean**	**SD**		
**McCully et al, 2005** [53]	0.47 mM muscle	0.07	0.36	0.06	p<.01	NR
**Ng et al, 1999** [58]	84.7 μg/g hair	73.3	46.8	28.9	p = .05	NR
**Rosborg et al, 2007** [66]	28.6 mg/l (median whole blood) 47.1 mg/l (median urine)	24.5–37.8 <25–189 (range)	28.2 60.5	23.2–37.2 <25–171	NS	NR
**Sakarya et al, 2011** [67]	x	x	x	x	x	*FIQ* *Pearson’s correlation coefficient*: 0.014 (NS)
**Sendur et al, 2008** [69]	x	x	x	x	x	*FIQ* *Pearson’s correlation coefficient*: -0.040 (NS)
**Manganese**						
**Kim et al, 2011** [46]	140 ng/g	80–260 (95%CI)	190	80–480	p = .029	NR
**Molybdenum**						
**Rosborg et al, 2007** [66]	0.6 μg/l (median)	<0.25–4.4 (range)	0.6	<0.25–5.7	NS	NR
**Phosphorus**						
**Kim et al, 2011** [46]	146 μg/g	116–183 (95%CI)	143	116–176	NS	NR
**Maafi et al, 2016** [49]	3.6 mg/dl	0.47	3.66	0.54	NS	NR
**Olama et al, 2013** [61]	3.55 mg/dl	0.12	3.6	0.16	NS	NR
**Türkyilmaz et al, 2010** [71]	3.2 mg/dl	0.4	3.3	0.5	NS	NR
**Ulusoy et al, 2010** [72]	3.54 mg/dl	0.56	3.57	0.46	NS	NR
**Polynutrient supplement**						
**Brouwers et al, 2002** [37]	Baseline CIS: 51.4 Follow up CIS: 48.6	4.2 7.4	51.3 48.2	3.6 7.6	NS	NR
**Potassium**						
**Jammes et al, 2011** [42]	3.92 mmol/l	0.12	3.99	0.08	NS	NR
**Kim et al, 2011** [46]	75 μg/g	25–219 (95%CI)	56	23–138	NS	NR
**Norregaard et al, 1994** [59]	3.25 mmol/l (median)	NR	3.9	NR	NS	NR
**Rosborg et al, 2007** [66]	926 mg/l (median urine)	205–3300 (range)	1410	378–5200	p = .013	NR
**Selenium**						
**Eisinger et al, 1997** [39]	83 ng/ml	17	87	12	NS	NR
**Kim et al, 2011** [46]	75 μg/g	25–219 (95%CI)	56	23–138	NS	NR
**Reinhard et al, 1998** [64]	Median: 70.8 μg/l	67.7–75.3 (95%CI)	76.8	73.4–81.6	p<.05	NR
**Study**	**Patients**		**Controls**		**Statistically significant**	**Linked to clinical parameter**
	**Mean**	**SD**	**Mean**	**SD**		
**Rosborg et al, 2007** [66]	117 μg/l (median whole blood) 18.4 μg/l (median urine)	77.6–207 5.5–55.7 (range)	105 23.5	66.4–137 2.3–52.2	p = .015 NS	NR
**Sendur et al, 2008** [69]	44.4 μg/dl	12.1	38.7	13.9	NS	*FIQ* *Pearson’s correlation coefficient*: 0.011 (NS)
**Sodium**						
**Jammes et al, 2011** [42]	138 mmol/l	0.5	140	0.4	NS	NR
**Kim et al, 2011** [46]	78 μg/g	31–195 (95%CI)	72	27–195	NS	NR
**Rosborg et al, 2007** [66]	1560 mg/l (median urine)	90.8–3705 (range)	1700	510–4790	NS	NR
**Zinc**						
**Eisinger et al, 1997** [39]	16.9 mmol/l	1.8	16.1	1.9	NS	NR
**Khalifa et al, 2016** [45]	75.87 μg/dL	5.5	93.21	11.94	p<.05	NR
**Kim et al, 2011** [46]	167 μg/g	120–232 (95%CI)	165	125–217	NS	NR
**La Rubia et al, 2013** [48]	66.48 ng/ml	18.82	106.8	22.41	p<.001	*PCS-12* *Pearson’s correlation coefficient*: 0.402 (p = .017)
**Maes et al, 2006** [51]	73.5 mg/dl	NR	87	NR	p = .0001	*Fibrofatigue scale* *Pearson’s correlation coefficient*: -0.039 (NS)
**Rosborg et al, 2007** [66]	6000 μg/l (median whole blood) 294 μg/l (median urine)	3720–9400 35.8–1230 (range)	5450 290	3900–7300 35.0–66.5	p = .026 NS	NR
**Sendur et al, 2008** [69]	102.8 μg/dl	24.7	77.2	31	p = .001	*FIQ* *Pearson’s correlation coefficient*: -0.106 (NS)

CI = confidence interval, CIS = checklist individual strength, FIQ = fibromyalgia impact questionnaire, FIQR = revised fibromyalgia impact questionnaire, NR = not reported, NS = not significant, PCS = physical component summary, SD = standard deviation, VAS = visual analogue scale, x = reported in meta-analyses.

#### Interventions

Five RCTs were included. The first RCT determined the effect of magnesium citrate treatment in combination with amitriptyline versus amitriptyline only, on FMS symptoms, over a period of 8 weeks [34]. They found that amitriptyline and magnesium supplementation was more effective on all measured outcomes than amitriptyline alone. The second RCT investigated the effect of a polynutrient supplement (containing several vitamins (including A, B, C, D, E), minerals (including calcium, magnesium) and (co)enzymes), on fatigue and physical activity of patients with CFS, over a period of 10 weeks [37]. They found no significant difference between the placebo and treatment group on any of the outcome measures. A third RCT examined vitamin C and E treatment combined with exercise versus exercise only, in FMS patients, over a period of 12 weeks [57]. Although both interventions lead to significantly higher vitamin A, C, and E serum levels, the FMS symptoms did not improve in both groups. Furthermore, the most recent RCT investigated the effect of vitamin D, on symptoms in CFS patients, over a period of 6 months [75]. Despite a statistically significant increase in vitamin D, they found no evidence of improvement in symptoms of fatigue or depression. Lastly, in the fifth RCT, cholecalciferol was administered for 20 weeks in FMS patients, with the dosage depending on patients calcifediol levels [74]. A significant treatment effect on intensity of pain was found in the treatment group versus placebo. No changes in somatization, depression and anxiety, physical and mental health, and FMS symptom severity were observed in both the treatment and placebo group.

#### Clinical parameters

All studies investigating vitamin A (n = 1) [67], vitamin C (n = 1) [67], ferritin (n = 2) [50,62], iron (n = 1) [50], and selenium (n = 1) [69], found no significant associations between vitamin and mineral status and clinical parameters in FMS patients (Table 3). Most studies investigating vitamin D (n = 6) found no significant associations between vitamin D and clinical parameters in CFS [75] and FMS [35,49,65,72,76] patients. However, two studies found significantly higher VAS-score for pain in patients with vitamin D levels <30 ng/ml compared to FMS patients with vitamin D levels of >30ng/ml [44,60]. Significant negative associations were found for vitamin E in plasma and fatigue in CFS patients (n = 1/2) [73], and serum and erythrocyte magnesium and fibromyalgia symptoms (n = 1/3) [34]. A significant positive association was found for serum zinc and somatic symptoms in fibromyalgia patients (n = 1/3) [48].

#### Vitamin and mineral status

All studies that investigated vitamin B12 (n = 1) [62], folic acid (n = 1) [62], iron (n = 2) [48,50], molybdenum (n = 1) [66], phosphorus (n = 4) [46,49,61,71,72] sodium (n = 3) [42,46,66], and iodine (n = 1) [66], and the majority of studies that investigated potassium (n = 3/4) [42,46,59], and selenium status (n = 4/5) [39,46,66,69] found no statistically significant difference between patients and controls (Table 3). In contrast, all studies that investigated vitamin B1 (n = 1/1) [54], and manganese (n = 1/1) [46], and the majority of studies that investigated vitamin A (n = 2/4) [39,67], found statistically significant lower serum values in patients versus controls. The majority of the studies that were not suitable for inclusion in the meta-analyses reported significantly lower vitamin E in patients versus controls (n = 3/4) [55,56,73]. Statistically significant results were found in the majority of the included studies investigating copper (n = 3/4) [46,48,66], ferritin (n = 4/5) [46,50,62,66], and zinc (n = 5/7) status [48,51,66,69]. However, the direction of the differences was equivocal for all three minerals: levels of copper were higher among patients in 3 studies and lower in 1, levels ferritin were higher among patients in 2 studies and lower in 2, and levels of zinc were lower in 3 studies and higher in 2.

### Meta-analysis

**Fig 2:**
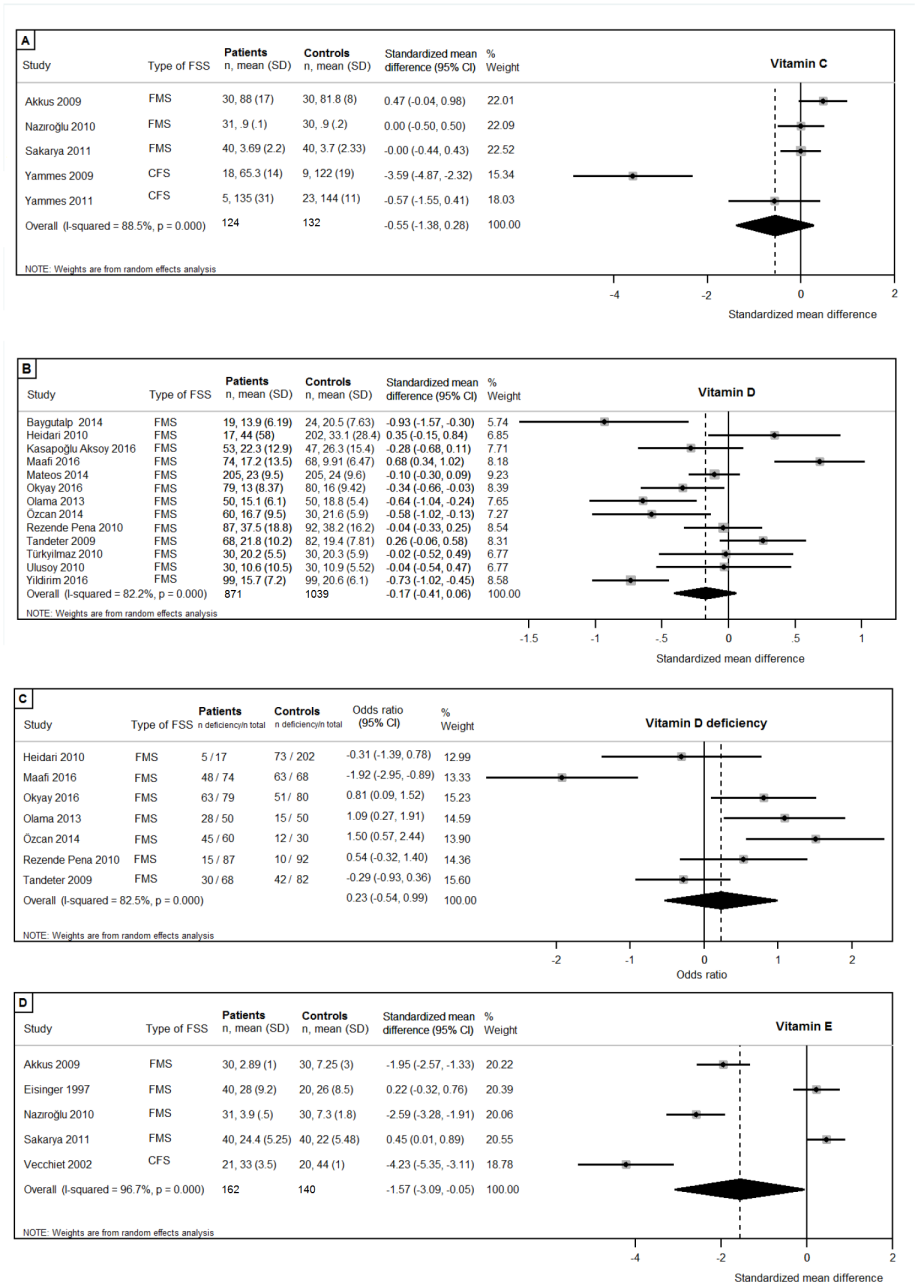
Forest plots of studies investigating vitamins. (A) Vitamin C; (B) Vitamin D; (C) Vitamin D deficiency (<20ng/ml); (D) Vitamin E.

**Fig 3:**
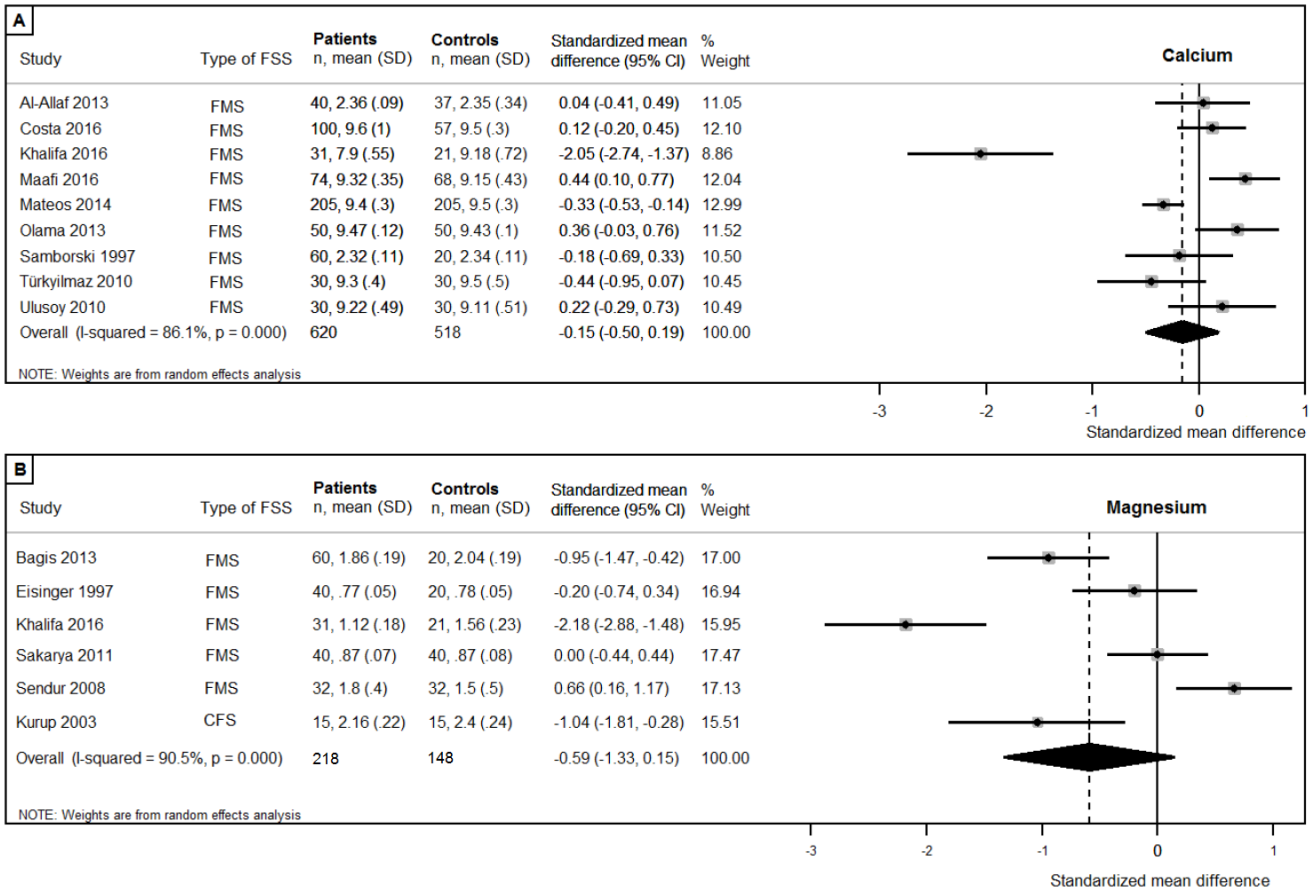
Forest plots of studies investigating minerals. (A) Calcium; (B) Magnesium.

Vitamin C, vitamin D, vitamin D deficiency (<20ng/ml), vitamin E (Fig 2), and the minerals calcium, and magnesium status, and were reported in more than five studies and were therefore investigated using meta-analysis (Fig 3). Meta-analysis revealed that circulating concentrations of vitamin E were lower in patients compared to controls (patients n = 162, controls n = 140; pooled SMD:-1.57, 95%CI:-3.09,-0.05; p = .042). No differences were found in patients compared to controls in circulating concentrations of vitamin C (patients n = 124, controls n = 132; pooled SMD:-0.55, 95%CI:-1.38,0.28; p = .19), vitamin D (patients n = 871, controls n = 1039; pooled SMD:-0.17, 95%CI:-0.41,0.06; p = .15), and vitamin D deficiency (patients n = 435, controls n = 604; pooled OR:0.23, 95%CI:-0.54,0.99; p = .17). There were no differences between patients and controls in circulating concentrations of the minerals calcium (patients n = 620, controls n = 518; pooled SMD:-0.15, 95%CI:-0.50,0.19; p = .38), and magnesium (patients n = 218, controls n = 148; pooled SMD:-0.59, 95%CI:-1.33,0.15; p = .12). All analyses revealed substantial to considerable heterogeneity in the effect sizes, as can be found in Fig 2.

#### Subgroup analyses

Subgroup analyses were performed including studies with more than half the maximum study quality score (>9 quality points), if more than three studies with a sufficient quality score were available. The additional analysis was not possible for magnesium, since only two studies achieved more than half of the maximum quality score. No differences in circulating concentrations of vitamin C (patients n = 93, controls n = 102, pooled SMD:-0.78, 95CI:-1.95, 0.39; p = .19) [32,42,43,67], vitamin D (patients n = 358, controls n = 376, pooled SMD:-0.07, 95%CI:-0.44,0.30; p = .71) [35,49,61,65,70–72], vitamin D deficiency (patients n = 121, controls n = 130; pooled OR:-0.12, 95%CI:-1.24,1.01; p = .84) [49,61,65,70], and calcium = (patients n = 184, controls n = 178; pooled SMD:0.18 95%CI:-0.18,0.54; p = .34) [49,61,71,72] were found. The significant difference in circulating concentrations of vitamin E between patients and controls disappeared when studies with low quality score were excluded (patients n = 91, controls n = 90, pooled SMD: -1.86, 95%CI:-4.28, 0.56; p = .13) [32,67,73].

Subgroup analyses were performed separately for the syndromes, when more than three studies were available per syndrome. Since vitamin D, vitamin D deficiency and calcium were only determined in FMS patients, additional subgroup analyses were possible for vitamin C, vitamin E and magnesium. No statistically significant difference between patients and controls was found in the three studies investigating circulating concentrations of vitamin C in FMS patients (patients n = 101, controls n = 100; pooled SMD:0.14, 95%CI:-0.16,0.44; p = .32). However, the heterogeneity was substantially lower (I^2^ = 13.3% versus 88.5% in the overall analysis including CFS patients), indicating a high consistency of studies’ results. The significant difference in circulating concentrations of vitamin E between patients and controls disappeared when the single CFS study was excluded (patients n = 141, controls n = 120; pooled SMD:-0.95, 95%CI:-2.41,0.50; p = .20. Lastly, no considerable differences were found in analyses of the five studies investigating circulating concentrations of magnesium in FMS patients (patients n = 203, controls n = 133; pooled SMD:-0.51, 95%CI:-1.34,0.32; p = .23).

#### Publication bias

**Fig 4:**
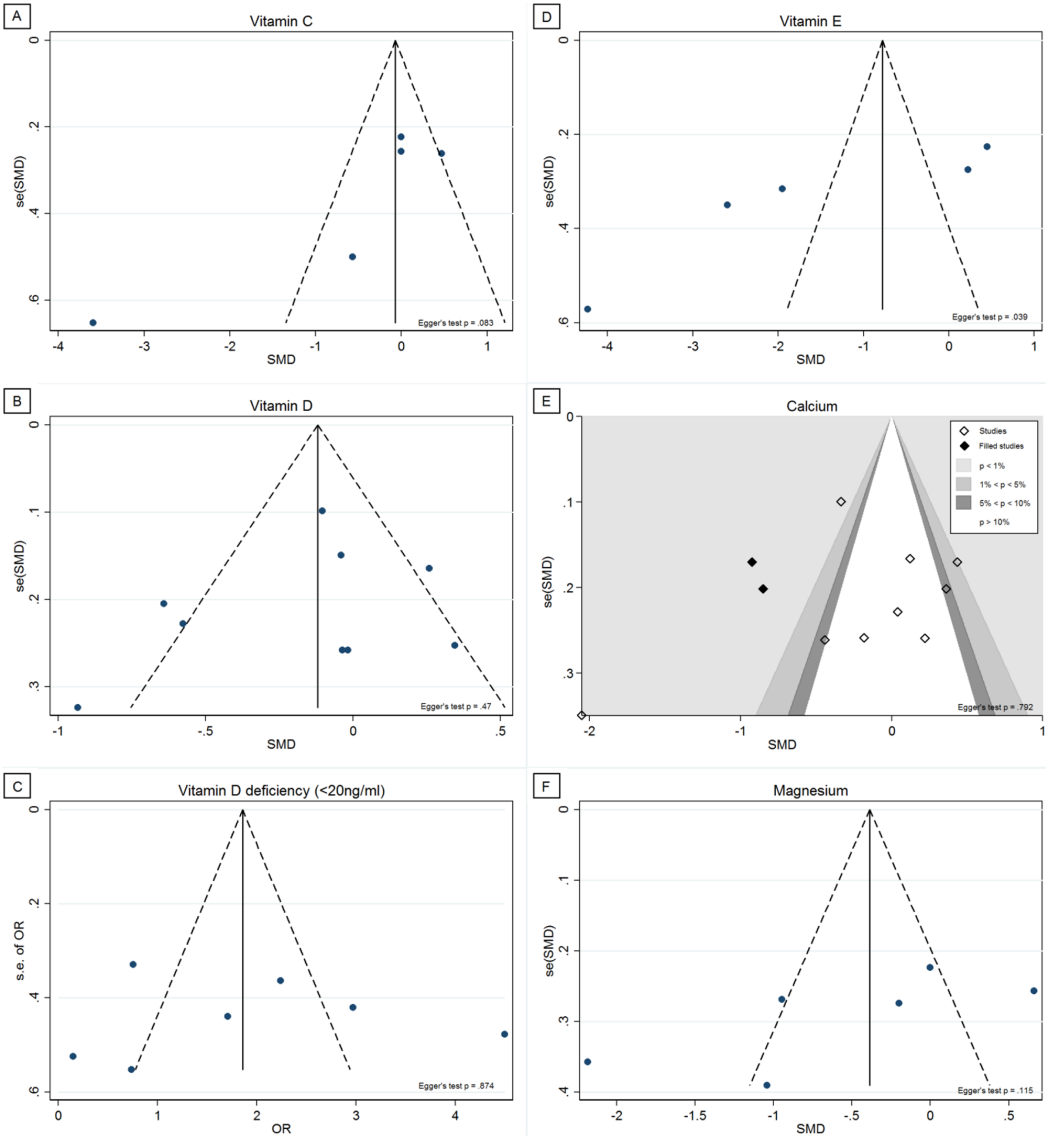
Funnel plots. (A) Vitamin C; (B) Vitamin D; (C) Vitamin D deficiency (<20ng/ml); (D) Vitamin E; (E) Calcium; (F) Magnesium.

Finally, we tested whether publication bias could have affected the results. Corresponding funnel plots can be found in Fig 4. Egger's test showed that there was significant funnel plot asymmetry in vitamin E (p = .039), with no significant asymmetry among the other analyses. Trimming was performed in the calcium studies using the Trim and Fill test, and the contour-enhanced funnel plot revealed two added studies in the statistically significant areas. No studies were trimmed or filled among the vitamin C, vitamin D, vitamin D deficiency, vitamin E, and magnesium studies, indicating absence of substantial publication bias.

## Discussion

We found little evidence to support our hypothesis that vitamin and mineral deficiencies play a role in the pathophysiology of both CFS and FMS, or that the use of nutritional supplements is effective in these patients. Poor study quality and considerable heterogeneity in most studies was found, which makes it difficult to reach a final conclusion. Consistent significant lower circulating concentrations were found repeatedly and in the majority of studies for vitamin A and vitamin E in patients compared to controls. However, the significant difference in circulating concentrations of vitamin E between patients and controls disappeared when excluding low quality studies. None of these or other vitamins and minerals have been repeatedly or consistently linked to clinical parameters. In addition, RCTs testing supplements containing these vitamins and/or minerals did not result in clinical improvements.

This review has several strengths. First, this is the first review focusing on vitamin and mineral deficiencies among CFS and FMS patients. We were able to give a clear overview of the current knowledge existing in literature. Second, we included only studies that examined CFS and FMS patients according to the official diagnostic criteria. We therefore have included relatively homogeneous groups of patients. Third, because we defined strict in- and exclusion criteria, e.g. patients should meet the official diagnostic criteria, or clinical cohorts must have an appropriate control group, poor quality studies were filtered out. Nevertheless, the vast majority of the included studies scored a quality score below a reasonable study quality. Fourth, enough studies that investigated similar vitamins or minerals were available, which made it possible to conduct six meta-analyses. Lastly, we had no language restrictions for the included abstracts or full text articles, which enabled us to include all relevant articles.

We must acknowledge that this study also has its limitations, which are mostly due to limitations in original studies on which this review was based. First, most studies were observational in nature. In general, observational studies have a lower validity than RCTs, and they are more susceptible to bias (e.g. selection and information bias) and confounding factors. Potential confounders were assessed in about half of the studies, but almost no studies adjusted their analyses for potential confounders. Consequently, the results of the current review may be affected by the methodological weaknesses that are accompanied by the observational study designs. Second, quality assessment revealed a poor study quality in the majority of studies. This demonstrates that substantial improvements can be made in terms of study quality, especially in specification of in- and exclusion criteria, presenting disease characteristics of the participants, making use of validated methods to assess vitamin and mineral status, to perform the vitamin and mineral assessments in duplicate, and, as mentioned earlier, to adjust analyses for potential confounders. Furthermore, a quality issue in research on CFS and FMS patients is that of careful selection of control groups. Our quality assessment showed that many included studies fell short because of the selection of the controls, which could result in inaccurate study results. Third, a problem that affects the validity of meta-analyses is the presence of publication bias. Funnel plots indicated the absence of publication bias in the majority of the meta-analyses. Trimming was performed among the calcium studies, and two “missing” studies were added, while no significant funnel plot asymmetry was present. However, trimming was performed in the statistically significant areas, which argues against the presence of publication bias. Although Egger’s test is preferred for more than 10 studies, it revealed significant funnel plot asymmetry in vitamin E, while no trimming was performed. It is therefore possible that the significant outcomes of vitamin E in patients are influenced by publication bias. Lastly, a substantial to considerable heterogeneity in most studies was found, which makes it difficult to reach a final conclusion about vitamin status in CFS and FMS patients.

This review reveals that very few RCTs have investigated the effect of vitamin and mineral supplementation versus placebo in CFS and FMS patients. Most published RCTs found no treatment effect of vitamin and mineral supplementation on clinical parameters. So, the evidence for beneficial effects of supplementation in CFS and FMS patients is not proportional to the large quantity of supplements that are used by these patients. Nevertheless, the industry of vitamin and minerals supplements is increasing, for example, Americans spend an estimated $36.7 billion each year on supplements [77]. This is important information, since the vitamins and minerals in these products are sometimes supplemented in doses high enough to cause side effects, for example gastric discomfort, insomnia, dizziness or weakness [17]. The vast majority of available studies concerned FMS patients. Several FMS studies investigated vitamin D, whereas most CFS studies have focused on vitamin E. Only one CFS study that investigated vitamin E was suitable for inclusion in the meta-analysis. It is remarkable that the significant difference of vitamin E between patients and controls disappeared when the single CFS study was excluded in the sensitivity analysis, while the studies that were not suitable for inclusion in the meta-analysis reported significant lower vitamin E concentrations in particularly CFS patients versus controls. Further research is needed to determine whether this may indicate that vitamin E levels are lower in CFS patients, but not in FMS patients. This systematic review and meta-analysis provides no further insights in whether the remaining vitamins and minerals differ between these two medical conditions.

We conclude that there is little evidence to support the hypothesis that vitamin and mineral deficiencies play a role in the pathophysiology of both CFS and FMS. Furthermore, the current literature on vitamins and minerals in CFS and FMS is of poor quality and stresses the need for well-performed intervention research, and large population-based and age-matched prospective studies in CFS and FMS, in order to gain more insight in the role of vitamins and minerals in the pathophysiology of CFS and FMS. According to our results, potential vitamins and minerals that should be further examined include vitamin A and vitamin E.

## Supporting information

S1 Table PRISMA checklist. (DOCX)

S1 Appendix Search strings. (DOCX)

S2 Appendix Quality tool to assess methodological quality of vitamin and mineral studies in CFS and FMS. (DOCX)

## Abbreviations

CFS: chronic fatigue syndrome
FMS: fibromyalgia syndrome
RCT: randomized controlled trial
