# Supplementary material for: Vitamin and mineral status in chronic fatigue syndrome and fibromyalgia syndrome: A systematic review and meta-analysis
Source: PLoS One. 2017 Apr 28;12(4):e0176631. doi: 10.1371/journal.pone.0176631 (PMC5409455; doi:10.1371/journal.pone.0176631)
Supplement: S1 Appendix — (DOCX) [file pone.0176631.s002.docx]

**S1 Appendix**

**PubMed**:

("Fatigue Syndrome, Chronic"[Mesh] OR "Fibromyalgia"[Mesh] OR functional somatic syndrome*[tw] OR chronic fatigue*[tw] OR postviral fatigue[tw] OR post-viral fatigue[tw] OR fatigue syndrome*[tw] OR myalgic encephalomyelit*[tw] OR royal free disease*[tw] OR fibromyalg*[tw] OR fibrositis*[tw])

AND

("Micronutrients"[Mesh] OR "Minerals"[Mesh] OR "Vitamins" [Pharmacological Action] OR vitamin*[tw] OR mineral*[tw] OR nutrient*[tw] OR micronutrient*[tw] OR retinol*[tw] OR Thiamin*[tw] OR Riboflavin*[tw] OR Niacin*[tw] OR Pantothenic*[tw] OR Pyridoxin*[tw] OR Biotin*[tw] OR Folic*[tw] OR folate*[tw] OR Cobalamin*[tw] OR Ascorbic*[tw] OR Calciferol*[tw] OR Tocopherol*[tw] OR Phylloquinone*[tw] OR Menaquinone*[tw] OR Calcium*[tw] OR Chromium*[tw] OR Chlorine*[tw] OR Copper*[tw] OR Fluoride*[tw] OR Iodine*[tw] OR Iron*[tw] OR Manganese*[tw] OR Magnesium*[tw] OR Molybdenum*[tw] OR Phosphor[tw] OR phosphorus[tw] OR phosphoric[tw] OR Potassium*[tw] OR Selenium*[tw] OR Sodium*[tw] OR natrium*[tw] OR Zinc*[tw])

NOT

(("Animals"[Mesh] NOT "Humans"[Mesh]) OR "Review" [Publication Type] OR systematic review [ti] OR animal* [ti] OR mouse[ti] OR mice[TI] OR rodent*[TI] OR rat[TI] OR rats[TI])

**EMBASE:**

'chronic fatigue syndrome'/exp OR 'fibromyalgia'/exp OR 'functional somatic syndrome':ab,ti OR 'chronic fatigue':ab,ti OR 'postviral fatigue':ab,ti OR 'post viral fatigue':ab,ti OR 'fatigue syndrome':ab,ti OR 'myalgic encephalomyelitis':ab,ti OR 'royal free disease':ab,ti OR fibromyalg*:ab,ti OR fibrositis*:ab,ti AND

('trace element'/exp OR 'mineral'/exp OR 'vitamin'/exp OR vitamin*:ab,ti OR mineral*:ab,ti OR nutrient*:ab,ti OR micronutrient*:ab,ti OR retinol*:ab,ti OR thiamin*:ab,ti OR riboflavin*:ab,ti OR niacin*:ab,ti OR pantothenic*:ab,ti OR pyridoxin*:ab,ti OR biotin*:ab,ti OR folic*:ab,ti OR folate*:ab,ti OR cobalamin*:ab,ti OR ascorbic*:ab,ti OR calciferol*:ab,ti OR tocopherol*:ab,ti OR phylloquinone*:ab,ti OR menaquinone*:ab,ti OR calcium*:ab,ti OR chromium*:ab,ti OR chlorine*:ab,ti OR copper*:ab,ti OR fluoride*:ab,ti OR iodine*:ab,ti OR iron*:ab,ti OR manganese*:ab,ti OR magnesium*:ab,ti OR molybdenum*:ab,ti OR phosphor:ab,ti OR phosphorus:ab,ti OR phosphoric:ab,ti OR potassium*:ab,ti OR selenium*:ab,ti OR sodium*:ab,ti OR natrium*:ab,ti OR zinc*:ab,ti)

NOT

('animal experiment'/exp OR ('animal'/exp NOT 'human'/exp) OR 'systematic review'/exp OR 'review'/exp OR 'systematic review':ti OR animal*:ti OR mouse:ti OR mice:ti OR rat:ti OR rats:ti OR rodent*:ti)

**Web of Knowledge:**

TS=(“chronic fatigue” OR “fatigue syndrome” OR “fibromyalgia” OR “postviral fatigue” OR “post viral fatigue” OR “myalgic encephalomyelitis” OR “royal free disease” OR “fibrositis”)

AND

TS=(micronutrient* OR nutrient* OR vitamin* OR mineral* OR retinol* OR thiamin* OR riboflavin* OR niacin* OR pantothenic* OR pyridoxin* OR biotin* OR folic* OR folate* OR cobalamin* OR ascorbic* OR calciferol* OR tocopherol* OR phylloquinone* OR menaquinone* OR calcium* OR chromium* OR chlorine* OR copper* OR fluoride* OR iodine* OR iron* OR manganese* OR magnesium* OR molybdenum* OR “phosphor” OR “phosphorus” OR “phosphoric” OR potassium* OR selenium* OR sodium* OR natrium* OR zinc*)

NOT

TI=(review* OR animal* OR "mouse" OR "mice" OR "rat" OR "rats" OR rodent*)

**PsycINFO:**

(DE "Fibromyalgia" OR DE "Chronic Fatigue Syndrome" OR TX (“chronic fatigue” OR “fatigue syndrome” OR “fibromyalg*” OR “postviral fatigue” OR “post viral fatigue” OR “myalgic encephalomyelitis” OR “royal free disease” OR “fibrositis”))

AND

(DE "Vitamins" OR DE "Ascorbic Acid" OR DE "Choline" OR DE "Folic Acid" OR DE "Nicotinamide" OR DE "Nicotinic Acid" OR TX (micronutrient* OR nutrient* OR vitamin* OR mineral* OR retinol* OR thiamin* OR riboflavin* OR niacin* OR pantothenic* OR pyridoxin* OR biotin* OR folic* OR folate* OR cobalamin* OR ascorbic* OR calciferol* OR tocopherol* OR phylloquinone* OR menaquinone* OR calcium* OR chromium* OR chlorine* OR copper* OR fluoride* OR iodine* OR iron* OR manganese* OR magnesium* OR molybdenum* OR “phosphor” OR “phosphorus” OR “phosphoric” OR potassium* OR selenium* OR sodium* OR natrium* OR zinc*))

NOT

TI (review* OR animal* OR "mouse" OR "mice" OR "rat" OR "rats" OR rodent*)
