# Supplementary material for: Vitamin and mineral status in chronic fatigue syndrome and fibromyalgia syndrome: A systematic review and meta-analysis
Source: PLoS One. 2017 Apr 28;12(4):e0176631. doi: 10.1371/journal.pone.0176631 (PMC5409455; doi:10.1371/journal.pone.0176631)
Supplement: S2 Appendix — (DOCX) [file pone.0176631.s003.docx]

**S2 Appendix**

**Key domain 1 (items 1-4): appropriate selection of participants.**

Patients have to meet the international criteria; the CDC case definition for CFS [1], and the ACR criteria for the classification of FMS [2, 3]. An appropriate control group has to represent the population from which the cases arose. Poor reporting of recruitment strategies and the recruitment of healthy controls from medical students or hospital staff may lead to selection bias and may threaten the validity of reported results [4].

Several somatic conditions, for example celiac disease, inflammatory bowel disease [5], diabetes [6], hypertension [7], cancer [8], and psychiatric conditions, such as anorexia or bulimia nervosa [9], and major depressive disorder [10], have been associated with the vitamin and mineral status. Additionally, medication use is important to consider, since use of several drugs, such as antidepressants [11], antihypertensive medication [12], corticosteroids [13], and anticonvulsants [14], are associated with vitamin or mineral status.

It is difficult to assess the length of disease as the formal diagnosis is typically made much later than the onset of somatic symptoms. However, in the initial phase of CFS or FMS other characteristics may be present than in the chronic course of CFS or FMS. Therefore, studies should report the central tendency of disease duration with an appropriate measure of distribution. Since there is currently no validated tool that indicates severity of CFS or FMS, measurements of for example somatic symptoms or quality of life should be reported as best available measure of severity.

**Key domain 2 (items 5-7): quantification of vitamin and mineral status.**

In addition to selection of participants, it is important to assess the reliability of vitamin and mineral quantification. We have chosen to incorporate three important requirements that a reliable analysis and data presentation should meet: presence of a detailed description of the analytical method or a statement of validation, measurements performed in duplicate and a clear mention of appropriate units and dispersion measures.

Validation was assumed when explicitly mentioned in the methods; analyses which were not stated as validated were also classified as such. Vitamin and mineral data were regarded as appropriate when reported in conventional or Système International (SI) units, e.g. concentration as unit of amount – mole, gram or international unit (IU) – per unit of volume – liter.

**Key domain 3 (items 8 and 9): appropriate control for confounding.**

There are potential confounders in the relationship between vitamin and mineral status and CFS or FMS. Age [15], sex [15,16], smoking [15,17], diet [15,18], body mass index [11] socioeconomic status [19], and psychiatric morbidity, are associated with the vitamin and mineral status. Age, sex, smoking, diet, body mass index, socioeconomic status [20-22], and psychiatric morbidity [23], are also associated with CFS and FMS. Therefore, these variables were included in the quality assessment tool.

**Quality tool to assess methodological quality of vitamin and mineral studies in CFS and FM**

**Appropriate selection of participants**

(1) Has the disease of the cases been reliably assessed and validated?

According to international criteria by a physician (2)

According to international criteria, assessor not clearly established (1)

Self-report or not clearly stated (0)

(2) Have all controls been recruited from the same population as the cases?

Same control population as cases (2)

Selected population, such as hospital staff or students (1)

Not clearly stated (0)

(3) Is the population defined with in- and exclusion-criteria?

Medication use, somatic morbidity, psychiatric morbidity, 3 stated (2)

Medication use, somatic morbidity, psychiatric morbidity 1-2 stated (1)

None stated or not clearly stated (0)

(4) Are disease characteristics presented (length and severity of CFS or FM)?

Duration of disease and severity of disorder is stated (2)

Only duration or only severity is stated (1)

None stated (0)

**Appropriate quantification of vitamin and mineral status**

(5) Are methods for assessment of vitamin and mineral status clearly stated?

Validated measurement tool (2)

Non-validated measurement tool, but the tool is available or described (1)

No description of the measurement tool (0)

(6) Was quantification performed in duplicate?

Yes (2)

No (0)

(7) Is outcome vitamin or mineral status clearly described and presented?

Appropriate units (absolute units or normalized units) and measures of dispersion stated (2)

Only appropriate units but no measures of dispersion stated (1)

Outcome not clearly stated (0)

**Appropriate control for confounding**

(8) Are potential confounders assessed ^a^?

Age, sex, smoking, diet, body mass index, socioeconomic status, psychiatric morbidity, medication use, 5–8 stated (2)

Age, sex, smoking, diet, body mass index, socioeconomic status, psychiatric morbidity, medication use, 3–4 stated (1)

Age, sex, smoking, diet, body mass index, socioeconomic status, psychiatric morbidity, medication use, 1–2 or none stated (0)

(9) Are the analyses adjusted for potential confounders ^b^?

Age, sex, smoking, diet, body mass index, socioeconomic status, psychiatric morbidity, medication use, 5–8 stated (2)

Age, sex, smoking, diet, body mass index, socioeconomic status, psychiatric morbidity, medication use, 3–4 stated (1)

Age, sex, smoking, diet, body mass index, socioeconomic status, psychiatric morbidity, medication use, 1–2 or none stated (0)

^A^ In case of exclusion at item 3, consider confounder as assessed.

^B^ In case of exclusion at item 3 or no significant difference between cases and controls at item 7 consider confounder as adjusted for.

**References**

1. Fukuda K, Straus SE, Hickie I, Sharpe MC, Dobbins JG, Komaroff A. The chronic fatigue syndrome: A comprehensive approach to its definition and study. Ann Intern Med. 1994;121(12):953-9.

2. Wolfe F, Smythe HA, Yunus MB, Bennet RM, Bombardier C, Goldenberg DL et al. The american college of rheumatology 1990 criteria for the classification of fibromyalgia. Arthritis & Rheumatism. 1990;33(2):160-72.

3. Wolfe F, Clauw DJ, Fitzcharles M, Goldenberg DL, Katz RS, Mease P, et al. The american college of rheumatology preliminary diagnostic criteria for fibromyalgia and measurement of symptom severity. Arthritis care & research 2010;62(5):600-10.

4. Lee W, Bindman J, Ford T, Glozier N, Moran P, Stewart R, et al. Bias in psychiatric case-control studies: Literature survey. Br J Psychiatry. 2007;190:204-9.

5. Jahnsen J, Falch J, Mowinckel P, Aadland E. Vitamin D status, parathyroid hormone and bone mineral density in patients with inflammatory bowel disease. Scand J Gastroenterol. 2002;37(2):192-9.

6. Walter RM,Jr, Uriu-Hare JY, Olin KL, Oster MH, Anawalt BD, Critchfield JW, et al. Copper, zinc, manganese, and magnesium status and complications of diabetes mellitus. Diabetes Care. 1991;14(11):1050-6.

7. Russo C, Olivieri O, Girelli D, Faccini G, Zenari ML, Lombardi S, et al. Anti‐oxidant status and lipid peroxidation in patients with essential hypertension. J Hypertens. 1998;16(9):1267-71.

8. Ames BN, Wakimoto P. Are vitamin and mineral deficiencies a major cancer risk? Nature Reviews Cancer. 2002;2(9):694-704.

9. Setnick J. Micronutrient deficiencies and supplementation in anorexia and bulimia nervosa A review of literature. Nutrition in Clinical Practice. 2010;25(2):137-42.

10. Morris MS, Fava M, Jacques PF, Selhub J, Rosenberg IH. Depression and folate status in the US population. Psychother Psychosom. 2003;72(2):80-7.

11. Aasheim ET, Hofso D, Hjelmesaeth J, Birkeland KI, Bohmer T. Vitamin status in morbidly obese patients: A cross-sectional study. Am J Clin Nutr. 2008;87(2):362-9.

12. Jacques PF, Bostom AG, Wilson PW, Rich S, Rosenberg IH, Selhub J. Determinants of plasma total homocysteine concentration in the framingham offspring cohort. Am J Clin Nutr. 2001;73(3):613-21.

13. Searing DA, Zhang Y, Murphy JR, Hauk PJ, Goleva E, Leung DY. Decreased serum vitamin D levels in children with asthma are associated with increased corticosteroid use. J Allergy Clin Immunol. 2010;125(5):995-1000.

14. Holick MF, Siris ES, Binkley N, Beard MK, Khan A, Katzer JT, et al. Prevalence of vitamin D inadequacy among postmenopausal north american women receiving osteoporosis therapy. The Journal of Clinical Endocrinology & Metabolism. 2005;90(6):3215-24.

15. Galan P, Viteri F, Bertrais S, Czernichow S, Faure H, Arnaud J, et al. Serum concentrations of β-carotene, vitamins C and E, zinc and selenium are influenced by sex, age, diet, smoking status, alcohol consumption and corpulence in a general french adult population. Eur J Clin Nutr. 2005;59(10):1181-90.

16. Dhonukshe-Rutten RA, Lips M, de Jong N, Chin A Paw MJ, Hiddink GJ, van Dusseldorp M, et al. Vitamin B-12 status is associated with bone mineral content and bone mineral density in frail elderly women but not in men. J Nutr. 2003;133(3):801-7.

17. Brot C, Jorgensen NR, Sorensen OH. The influence of smoking on vitamin D status and calcium metabolism. Eur J Clin Nutr. 1999;53(12):920-6.

18. Herrmann W, Schorr H, Purschwitz K, Rassoul F, Richter V. Total homocysteine, vitamin B(12), and total antioxidant status in vegetarians. Clin Chem. 2001;47(6):1094-101.

19. Shahar D, Shai I, Vardi H, Shahar A, Fraser D. Diet and eating habits in high and low socioeconomic groups. Nutrition. 2005;21(5):559-66.

20. van’t Leven M, Zielhuis GA, van der Meer, Jos W, Verbeek AL, Bleijenberg G. Fatigue and chronic fatigue syndrome-like complaints in the general population. The European Journal of Public Health. 2010;20(3):251-7.

21. Branco JC, Bannwarth B, Failde I, Abello Carbonell J, Blotman F, Spaeth M, et al. Prevalence of fibromyalgia: A survey in five european countries. Semin Arthritis Rheum. 2010.39(6):448-53.

22. Rusu C, Gee M, Lagacé C, Parlor M. Chronic fatigue syndrome and fibromyalgia in canada: Prevalence and associations with six health status indicators. Health Promotion. 2015;35(1).

23. Janssens KA, Zijlema WL, Joustra ML, Rosmalen JG. Mood and anxiety disorders in chronic fatigue syndrome, fibromyalgia, and irritable bowel syndrome: Results from the LifeLines cohort study. Psychosom Med. 2015;77(4):449-57.
